# Monthly Summary of Medical Journalism

**Published:** 1860-03

**Authors:** O. C. Gibbs

**Affiliations:** Frewsburg, N. Y.


### ECLECTIC DEPARTMENT,

### AND SPIRIT OF THE MEDICAL PERIODICAL PRESS.

*Monthly Summary of Cotemporary Medical Journalism.* By O. C. Gibbs, M.D., Frewsburg, N. Y.

*A New Cure for Ingrowing Nail.*—In the *Boston Medical and Surgical Journal* for December 29th, Dr. N. Gilman gives a new plan of treating ingrowing nail, for which he claims prompt and satisfactory results. The treatment which he advises he says he has put to the test for twenty years, without failure. It is simply to cauterize the part with hot tallow. He says: "The patient on whom I first tried this plan was a young lady who had been unable to put on a shoe for several months, and decidedly the worst case that I have ever seen. The disease had been of long standing. The edge of the nail was deeply undermined, the granulations formed a high ridge, partly covered with skin, and pus constantly oozed from the root of the nail. The whole toe was swollen, and extremely tender and painful. My mode of proceeding was this: I put a very small piece of tallow in a spoon, and heated it over a lamp till it became very hot, and dropped two or three drops between the nail and the granulations. The effect was almost magical. Pain and tenderness were at once relieved, and in a few days the granulations were all gone, the diseased parts dry and destitute of feeling, and the edge of the nail exposed so as to admit of being pared away without any inconvenience. The cure was complete, and the trouble never returned. I have tried this plan repeatedly since, with the same satisfactory results."

*Asphyxia Neonatorum.*—In the *Lancet and Observer* for January, Dr. A. T. Keyt, of Cincinnati, has an article on the treatment of asphyxia of new-born children, in which he compares the old, or "mouth-to-mouth" treatment, with the "Ready Method" of Dr. Marshall Hall. Dr. Keyt prefers the old method, and with a fair show of reason. "It may be remembered that the case of the asphyxiated new-born child is not just parallel with that of the asphyxiated adult. The first has never respired. The chest has never been expanded; the air-vesicles have never been opened. The chest and lungs, then, do not possess that elasticity or resilience which would be so important an element in successfully carrying on the 'rotation process.' It would be difficult to understand how, under it, the first expansion of the lungs could take place; when the child is turned on its face, the lungs being already compressed, the capacity of the chest could be thereby but little, if any, diminished; and when turned upon the side, and a little beyond, as directed, it could be but little, if any, increased."

In reporting cases of comparative trial, in one he says: "*At least one hour elapsed before the child gave a gasp*, and two hours before it could be left to do its own breathing. My dependence was upon the *mouth-to-mouth* process: by it I found no difficulty in controlling the circulation! It seemed as though the heart's action might have thus been maintained indefinitely. The 'Marshall Hall Method' was tried, but the results were negative; under it pallor and lividity would return to the surface, and the circulation grow gradually more and more feeble, until the heart's action would plainly have soon ceased, had it not been timely aroused by a more *ready* and *efficient* method. Several times did I alternate the new method with the old, and just as often did I witness the same striking contrast of phenomena."

This is an interesting subject, and we must confess to a liking for the old rather than the new method in asphyxiated new-born infants. Where the child has a beating heart at birth, by judicious and persevering efforts respiration and resuscitation can usually be established. Where the lungs have never been expanded, we cannot see how the "rotation process" of Dr. Hall can be of much service, and we are glad of this opportunity of cautioning our younger readers against placing too much reliance upon it in such cases—inflate with your own breath, and then you may know that the inflation is being effected.

*Treatment of Prolapsus of the Funis.*—Prolapsus of the funis is not an uncommon accident, and, without appropriate treatment, it is one that often results unfortunately to the life of the child and the hopes of the mother. Professor Mendenhall, of Cincinnati, applauds the treatment of this accident by position of the mother: successful cases have been reported. In the *Lancet and Observer* for January, he reports another case converted, in a few moments, by this method, into a simple case of labor. He places the woman upon her breast and knees, in which position the funis is readily replaced. The position may be maintained, if need be, until the presenting part occupies the pelvic strait. It is probable that the position need not be long maintained. Prof. Mendenhall concludes his paper with the following remark: "In view of the frequent fatality to the child of this complication, I deem a knowledge of its proper treatment a matter of great importance. I think with this knowledge that few, if any, cases ought to result unfavorably to the child, and a resort to turning the child is seldom, if ever, necessary."

*Gelsemin.*—In the *Medical Press* for January 2d, Dr. B. Keith, of New York, has the following upon gelsemin, which article he says he has used daily for the last eight years: "For controlling fevers of every type and grade; to arrest hæmorrhage from the lungs, stomach, bowels, uterus, and urinary organs; in dysentery and bowel complaints; in spermatorrhœa, amaurosis, deafness, catarrhal affections, hay-fever, I have used the gelsemin successfully. A single half grain has arrested hæmorrhage from the lungs, when all other remedies known to me had failed. While experimenting with it to ascertain its power for arresting hæmorrhage, I gave to a lady who had been confined two days previous, one and a half grains during twenty-four hours, which amount completely arrested the hæmorrhage. I administered two grains, during the course of thirty-six hours, to a lady who had been suffering from uterine hæmorrhage for two months, and that small quantity completely stopped the flow. So effectual is it in this form of hæmorrhage, that I consider it quite a specific. In dysentery and bowel complaints, I consider it the most valuable article in the Materia Medica. From one-tenth to one-eighth of one grain administered after each discharge, will shortly stop all hæmorrhage and traces of the disease." * * * "In dry coughs, dependent upon irritation of the throat, it is the most prompt agent I have ever used. In nausea and vomiting I have used it, many cases yielding to a single dose of one-fourth of one grain."

*Lupulin in Delirium Tremens.*—In the *Medical and Surgical Reporter* for December 31st, Dr. D. S. Gloninger, of Philadelphia, has an article upon the use of lupulin in delirium tremens. Of the treatment he says: "Our only indication is sleep, and that must be brought about with the least risk to the sufferer. Have we that which will effect so desirable an end? In lupulin we have that which answers every indication; it is safe; may be given *ad libitum* without danger; a table-spoonful, if you please, every hour until sleep is produced. We have given as much as six pounds before its narcotic effects followed, and would have had no hesitation in giving four times the quantity, were six insufficient."

The above quotation justifies no other inference than that pure lupulin was used; yet, in the succeeding report of a case, it is stated that a tincture of lupulin, made with *pure brandy*, was used. It will readily be seen that there is a difference between *six pounds* of lupulin and six pounds of tincture. If the latter was used, it is quite probable that the brandy had some share in the cure. Will the Doctor please give further particulars?

*Chloroform and Cod-Liver Oil in Diphtheritis.*—In the *Boston Medical and Surgical Journal* for January 5th, Prof. E. S. Cooper, of San Francisco, has an article upon the above subject. In this affection he thinks the use of the probang injurious. He says, "My treatment is as follows:

| | | |
|---|---|---|
| R.—Chloroform, | ℥iij. | |
| Ol. jec. anseli, | ℥xij. | |
| Spts. terebinth., | ℥ij. | M. |

Apply freely all over the neck, breast, and abdomen, upon flannels covered with oil silk. This I keep on constantly during the continuance of the disease, and for eight or ten days after the patient has sufficiently recovered to walk about. The object of continuance is to prevent relapses, which are very frequent and fatal, without some prevention is used. And this is what is wanted in these cases. Internally, I direct the following to be administered:

| | | |
|---|---|---|
| R.—Ext. glycyrrh., | ℥iij. | |
| Acaciæ gum., | ℥j. | |
| Antimon. tart., | gr. j. | |
| Sacch. alb., | ℥ij. | |
| Aquæ, | ℥xviij. | M. |

Give a wine-glassful every two hours to a young child, say two years old, and increase in proportion to age. I have had as much, if not more, satisfaction in the results of the treatment of diphtherite on the foregoing plan than in anything occurring in my professional life besides. I therefore recommend it with confidence to the medical profession. I have tried it with nearly the same success in scarlatina. During the course of treatment I do not give patients a particle of anything else; not a drop of water, nor the least nourishment, save what is in the medicine."

With this treatment in diphtheritis he says, "Of thirty-one patients I have lost but one; and in that case the patient had been sick for several days, and died about eight hours after I first saw him."

This is certainly a very satisfactory result. For convenience of administration, especially to young children, we consider the size of the dose an inconvenience; and the absence of nourishment, especially in scarlet fever, we cannot believe adds efficacy to the treatment.

*Puerperal Convulsions.*—In the *New York Monthly Review, &c.*, for January, Prof. White, of Buffalo, reports a case of puerperal convulsions of great severity, treated without bloodletting, but quite successfully, with chloroform, &c. "Dr. White thinks puerperal convulsions a disease *sui generis*, not apoplectic, nor epileptic; hence, bleeding is seldom necessary." * * * "The puerperal convulsion is, no doubt, caused by some remote uterine irritation, perhaps uræmia, though the latter is not constant. This condition tends to develop convulsions. His theory is, that we should give chloroform and anodynes to relieve this irritable state of the system, to be followed with croton oil as a counter-irritant. This is easily administered, and acts as a powerful revulsive. He thought we should never arrive at a correct theory, or satisfactory treatment of this disease, until we change our notions in relation to the character of the seizures." We copy the above opinions with pleasure, corresponding, as they do, exactly with our own. We believe more women have died in childbed from the lancet than from convulsions. Cases of puerperal convulsions probably do occur, requiring the abstraction of blood; upon this point we will not call in question the universal judgment of the profession, but we may be permitted to say that such we have never seen. In eleven years' experience we have never bled a case of puerperal convulsions, nor lost a woman in childbed. Eschewing the charge of egotism, we attribute this favorable issue rather to the casualties of good luck than to legitimate sequences of peculiarities of treatment.

*Bronchitis.*—In the *Medical and Surgical Reporter* for January 7th, Dr. J. R. McClury has an article upon bronchitis. In regard to treatment in the acute form, after bloodletting, if indicated, he advises the following:

| | | |
|---|---|---|
| "℞.—Nitratis potassæ, | ʒij. | |
| Tartari emetici, | gr. ij. | |
| Tilden's verat. viride, | f. ʒj. | |
| Aquæ font., | f. ℥viij. | M. S. |

Take a table-spoonful every three hours in a tea-cup of flaxseed tea."

In cases of chronic bronchitis the following is advised:

| | |
|---|---|
| "℞.—Potassii ferrocyanuret, | f. ʒiv. |
| Vin. colchici sem., | f. ℥j. |
| Tilden's verat. viride, | f. ʒij. |
| Aquæ font., | f. ℥j. M. S. |

Take from 20 to 30 drops three or four times per day."

In cases of a scrofulous character, or in scrofulous constitutions, the following is recommended:

| | |
|---|---|
| "℞.—Syrup sarsap., | f. ℥viij. |
| Iodide potassium, | ʒij. |
| Fowler's Solution, | f. ʒij. |
| Tilden's verat. viride, | f. ʒjss. M. S. |

Take a tea-spoonful three times per day."

We believe it was Prof. G. B. Wood, of Philadelphia, who first advised Fowler's Solution in this form of bronchitis, and we have no doubt of its utility in many cases.

In cases accompanied with great debility, Dr. McClury advises cod-liver oil in usual doses, and the following:

| | |
|---|---|
| "℞.—Mist. ferri comp., | f. ℥vj. |
| Tilden's verat. viride, | f. ʒij. M. S. |

Take a tea-spoonful three times per day."

Dr. McClury thinks opium objectionable in all cases of bronchitis. He thinks the veratrum viride is equally efficacious as a sedative, and far less objectionable.

*Belladonna as an Antigalactic.*—In preceding numbers of our *Summary*, we have accumulated evidence of the power of belladonna to control the secretion of milk. Our own experience has also been already given. In the *Medical and Surgical Reporter* for January 7th, Dr. G. E. Galen reports three cases in which the secretion of milk was arrested, and abscess prevented by the use of this article.

He says: "I have tried it in a number of cases, and never yet met with a single failure. What peculiar action the belladonna has in arresting the secretion of the mammary gland, I do not know, but that it does so, if used continuously, and in sufficient proportions, I am certain."

*Bronchocele.*—In the *N. A. Medico-Chirurgical Review*, for January, Dr. O. B. Knode, of St. Joseph, Missouri, reports the cure of a severe case of bronchocele. A puncture was made, and five pints of fluid were withdrawn. It was now firmly strapped with adhesive plaster, and five grains of iodide of potassium were administered three times a day. In about fifteen months the cure was complete.

*Stomatitis Materna.*—In the last number of our *Summary*, we gave Dr. J. C. Reeve's plan of treating this troublesome affection with the compound syrup of the phosphates. In the *N. A. Medico-Chirurgical Review*, for January, Dr. E. J. Fountain, of Davenport, Iowa, has an article upon the same treatment of this disease. He has treated four well-marked cases of stomatitis materna with the compound syrup of the phosphates, with the happiest results. He says: "These cases impressed my mind with the belief that, in the syrup of the phosphates, freely administered, we had a pleasant and efficient remedy for the fulfillment of the most important indication. In simple and mild cases it has appeared to be the only remedy needed; but in those of a more aggravated character it cannot be depended upon as a specific, but must be aided by other treatment appropriate to particular symptoms. In the case last referred to, I increased the strength of the syrup, by the addition of an extra quantity of the phosphate of iron and phosphate of lime, giving to the patient five grains of each with every dose, and finally administered in connection with cod-liver oil."

*Inversion of the Uterus.*—Our readers will doubtless remember our extract from Prof. Bedford's Lectures, in our *Summary* for February, upon the reduction of uterine inversion. It will be remembered that he advised the reduction to be made before removing the placenta. In the *N. A. Medico-Chirurgical Review*, for January, Dr. Wm. Irvin, of Pennsylvania, reports a case of inversion and reduction, teaching, by inference, an opposite doctrine. Immediately after inversion, he says, "I severed the cord, and made several unsuccessful attempts to reduce the uterus, with the placenta still adhering. The hæmorrhage became very alarming, and the woman was sinking, when I remembered Prof. Meigs' injunction to remove the placenta and return the organ, by pressure upon its fundus. I therefore detached it as quickly as possible, and had the satisfaction of finding that the bleeding was much diminished." The uterus was soon reduced, and the patient made a good recovery.

*Miasmatic Hæmaturia.*—In our *Summary* for February, we made reference to the paper of Dr. Brickell upon this subject. The article referred to, and the editorial remarks of one of the editors of the *New Orleans Medical News and Hospital Gazette*, have called forth two other articles upon this subject, which articles will be found in the January issue of the above-mentioned journal. One of the papers referred to is by Dr. J. C. Cummings, of Monroe, La. Dr. Cummings differs from Dr. Brickell in regard to the nature of the hæmaturia. He thinks it is not *miasmatic*, and one of his reasons for this opinion is, that "most cases have occurred on the oldest plantations in this parish." Another reason given is, that "it is a disease entirely unknown here until within the last three or four years." He says that during the past summer he has seen and treated five cases; three of the five cases died. Tannin was a favorite remedy with Dr. Cummings.

We confess to a preference for the opinions of Dr. Brickell, and think it is quite probable that, had Dr. Cummings treated his cases with liberal doses of quinine, opium, and perhaps tannin, the result might have been different.

The other paper in the January number of the *News and Gazette* is by Dr. Marsh, of Port Hudson. Whereas Dr. Cummings considers the disease of quite recent origin, Dr. Marsh says, "I was not aware that this disease was considered more rare in malarious districts than other forms of nondescript intermittents, appearing under the garb of pneumonia, dysentery, gastritis, &c. He regards the hæmorrhage as the result of an intermittent engorgement, and says, "Arrest the intermittent, and the hæmaturia, pneumonia, flux, or any other disease it may choose as a mask, ceases." He says he saw three cases last August, all of which were treated with quinine, counter-irritation, and soon recovered.

We cannot conclude our notice of the paper of Dr. Marsh without referring to an idea that we consider erroneous. He says, "When I read in the medical journals of the day of the successful treatment of pneumonia and other inflammatory diseases by quinine, I feel confident the writers are mistaken in their diagnosis; that they are treating a true and genuine intermittent, disguised in all the habiliments of idiopathic inflammation."

We have treated quite a number of cases of pneumonia with quinine, opium, and ipecac, and the "journals of the day" contain the record of such experience. It is easy to assert that our diagnosis is in error—and we shall attempt no self-defence upon this point—but when he says we "are treating true and genuine intermittents, disguised, &c." we enter a demurrer; for an intermittent or remittent fever was *never known to originate* in the present range of our practice, and where we have found quinine, qualified as above, so serviceable in pneumonia. It may be well to observe here, that it is not every case of pneumonia that is best treated with quinine. The pulse, tongue, and the general condition of the patient are the guides to a correct indication.

*Acetic Acid in Erysipelas.*—In the *Atlanta Medical and Surgical Journal*, Dr. Wm. Hauser has an article upon the above subject. He regards erysipelas as the result of a super-alkaline condition of the system, and thinks acetic acid the appropriate treatment. In fact, he is of opinion that the benefits following the use of the muriated tincture of iron are mainly due to the acid character of the medicine.

We are not quite prepared to give full assent to Dr. Hauser's opinions, but the subject is evidently worthy of further observation.

*Iodine Injections in Lymphatic Tumors.*—In the *Chicago Medical Journal*, for January, Prof. Daniel Brainard reports a case of lymphatic tumor, successfully treated with iodine injections. The tumor was situated immediately above the clavicle, behind the sterno-mastoid muscle. It was tapped, and twelve ounces of fluid drawn out. The tumor was then injected with a "small quantity of a solution composed of iodine, one scruple; iodide of potassium, one drach.; to one ounce of water. A small quantity remained." A perfect cure resulted.

*Treatment of Indolent Ulcers.*—In the *Chicago Medical Journal*, for January, Prof. Brainard has the following upon the treatment of indolent ulcers: "During the last three years nearly all the cases of indolent ulcers, entered under our care, to the U. S. Marine Hospital, have been treated by the vapor of iodine. The result is very satisfactory in nearly all cases; more so, by far, than that obtained by any other single method. Its advantages are conceived to be these:

1. Cleanliness and facility of application.

2. Rapidity of cicatrization.

3. Destruction of the odor of the ulcer. Iodine acts as a disinfectant, like chlorine.

The manner of using it is as follows:

1. Dress the ulcer with simple cerate, spread on lint.

2. Take from one to four grains of iodine, according to the size and degree of indolence of the ulcer, folded in several layers of lint, and place it on the ulcer, over the first layer.

3. Cover this with a piece of oiled silk and tin-foil, which should be large enough to extend beyond the edges of the ulcer. This is to prevent rapid vaporization, and it should be secured by a roller. The warmth of the member speedily vaporizes the iodine, and a sensation of warmth is perceived by the patient on the ulcerated surface. If applied in too large quantity, or too directly on the surface, the iodine acts as an escharotic. Care is therefore required in this respect."

*Treatment of Intermittents.*—In the *Nashville Journal of Medicine and Surgery*, for January, Prof. W. K. Bowling has a very able article upon the treatment of malarious fevers in general, and intermittents in particular. It is impossible to give a complete summary of Prof. Bowling's able and eloquent article without making larger quotations than we have space for at present.

The opinions of Prof. Bowling are in accordance with those which we have long entertained, and feebly advocated for years, and are, consequently, of peculiar interest to us. We are confident that any physician that makes them his guide in practice will never have occasion to regret it. Our readers will remember that we gave a summary of W. A. Brown's article in a former number of the MONTHLY. Dr. Brown is of opinion that the complications and sequelæ are mainly due to neglect or inefficient treatment. Prof. Bowling, also, believes, and with this idea we have long coincided, that, if timely administered, quinine is adequate to the prevention or cure of all malarious diseases. He who spends a week or two in bleeding, and vomiting, and purging, and giving calomel, will have no reason to rejoice at his success. In 1680, the immortal Sydenham said that bleeding and purging only prolonged the disease; in his own practice, he resorted immediately to the Peruvian bark, and he advised his professional brethren to do the same, declaring that he never found any ill consequences to follow its use. Sydenham did not believe that any preparatory treatment was necessary in simple intermittents, and Prof. Bowling says, "Our various classes in the University of Nashville know that I have always taught precisely the same doctrine. That I have there every year declared that simple intermittent fever could be 'cured' by quinine, and its return prevented by quinine, and quinine alone, in both cases. That six grains of quinine was a good medium dose (given stirred up in a little water) in this latitude, which ought to be increased as we go South towards the Gulf, or Southeast towards the sea-board. That three such doses were sufficient for the *coup-de-grâce* of simple intermittent. That if we have the selection of time to begin with it, we choose that nearest to the *last* paroxysm, the beginning of the sweating stage of that paroxysm being preferred. And this rule we obey, whether the type be quotidian, tertian, or quartan. I make the interval between these doses two hours, so that eighteen grains are taken in four hours." * * * "On the ninth day, counting from the last paroxysm, however well the patient may be, I insist that two more such doses of quinine be taken, and so two doses more every ninth day, one in the morning and one in the evening, for five consecutive periods of nine days each. He is now certainly relieved from the effects of his poison, and if he were ever after to stay away from those localities where it is generated, he would never have another attack."

The above fully corresponds with our idea. We have always resorted, in malarious disease, to quinine without delay, and on all suitable occasions have advised the same, *believing that the best preparatory treatment for intermittents and remittents is that which will immediately prevent a return of the paroxysms.* After such interruption, the secretions, if at fault, can be corrected at leisure. We have long since believed that relapses were seldom necessary if the remedy was sufficiently persevered in. Quinine is a *preventive* as well as a *cure*, and should be administered with that in view, after the apparent cure.

In *inflammatory intermittents*, Prof. Bowling says, "In this climate, a single moderate bleeding will alone, in a large majority of cases, reduce an inflammatory intermittent to the simple form, when, if quinine, in twelve-grain doses instead of six, be administered *immediately* after the bleeding, and be repeated at intervals of two hours, until thirty-six grains are taken, the disease is conquered at once."

Where, for any reason, the bleeding is deemed not advisable, we believe the same end may be secured by administering a full dose of opium with the quinine, from three to six hours before the chill is expected. We do not mean one grain, but from three to six grains. In congestive intermittents, if the case is seen very early, and before active inflammation is induced, we question whether the opium is not preferable to bloodletting.

Of quinine, Prof. Bowling says, "I have been in the habit of regarding the sulphate of quinine as the greatest of blessings vouchsafed by a beneficent Providence to afflicted man, and a defence of its eminent virtues has ever been to me a most pleasurable employment."

*Chlorate of Potash in Acute Rheumatism.*—In the *St. Joseph Journal of Medicine and Surgery*, for January, Dr. J. B. Snelson has an article on the treatment of acute rheumatism with chlorate of potassa. He reports three cases thus treated, with results quite satisfactory. The following is his formula for administration:

| | | |
|---|---|---|
| ℞.—Chlorate of potassa, saturated solution, | f. ℥vj. | |
| Tinct. veratr. virid., | ʒss. | M. |

Dose.—A table-spoonful three or four times a day, and ten grains of Dover's powder at bedtime. The trial is too limited for positive results; yet Dr. Snelson thinks "it holds out encouraging hopes that it may have superior claims as a remedy over that painful and troublesome disease."

*Cholera Infantum Cured by a Poisonous Dose of Fowler's Solution.*—In the January issue of the *St. Joseph Journal of Medicine and Surgery*, Dr. P. A. Chambers reports a singular case of the cure of a dangerous disease by a still more dangerous accident. A child two years old, laboring under cholera infantum, that had been for a long time ineffectually treated, and of which the Dr. had but little hopes of cure, had administered to it, by mistake, a tea-spoonful of Fowler's solution. The child vomited soon after, seemed to suffer great pain in its bowels, and on the following day commenced to purge blood. It was not seen by a physician until two days after, and then mucilaginous drinks constituted the treatment. In a few days it began to improve, and recovered from the effects of the poison and from the disease at the same time. Dr. Chambers says, "*Similia similibus curantur* is the only way I can explain the beneficial effects of the poison upon the previous disease." If the cure was effected upon Homœopathic principles, it must be admitted that the dose was not in accordance with Homœopathic practice.

*Hypophosphites in Phthisis.*—In the *Medical Press* for January 14th, is published a letter from Dr. J. J. Campbell, of Brooklyn, to Dr. J. Winchester, reporting the effects of the hypophosphites of lime and soda in his own case. The night-sweats soon ceased to trouble, and the nervous system so improved as to permit sound and refreshing sleep. He says, "When I commenced the use of this remedy, five weeks ago, I weighed only 147 lbs.; now I weigh 161 lbs., a slight increase over my usual weight. My appetite is good, I sleep well, and I feel as if I were going to live in spite of the formation of a cavity in the upper portion of my right lung." This is certainly an encouraging result of the new remedy. The hypophosphites are much more pleasant to take than cod-liver oil, and we hope the effects following its use may be a still more important improvement.

*Gastrotomy.*—Some of our readers may remember that about five years ago Dr. John Bell, of Wapello, Iowa, reported, in the *Iowa Medical and Surgical Journal*, the extraction of a bar of lead from the stomach—the case being operated upon by himself, and resulting in recovery. The report of the case is reproduced in the *Boston Medical and Surgical Journal* for January 19th. The bar of lead was $10\frac{3}{4}$ inches long, and weighed $9\frac{1}{4}$ ounces. The incision into the stomach was made on the left anterior side, and about parallel with the pylorus. The patient made a good recovery, and was discharged on the 15th day after the operation.

*Iodide of Potassium in Diseases of the Brain in Children.*—In the

*Boston Medical and Surgical Journal* for January 19th, is an article upon the above subject, by John Coldstream, M.D., &c, copied from the *Edinburgh Medical and Surgical Journal.* Dr. Coldstream says that for more than twenty years he has used the iodide of potassium in brain disturbances of children, particularly in hydrocephalus. He says, "The results I have obtained have been so much more decidedly favorable than those which I had been accustomed to see under the employment of depletion, calomel, and purgatives, that I have been surprised to find comparatively few references to the treatment of diseases of the head by this agent in the more recent works on the practice of medicine. I have met with but a small number of practitioners who seem to recognize it as a remedy of marked efficacy." Following the above remarks, Dr. Coldstream makes allusion to, and quotes the opinions of those who have spoken well of this remedy in this class of diseases.

In the summer of 1849, and consequently about eleven years ago, we commenced the treatment of a case that we diagnosticated to be one of scrofulous meningitis, in its early stages. The case recovered, and remains well up to the present time. We afterwards treated other cases in a similar manner, it is true not always with the same good results, but, at least, successful in the majority.

We were not then aware that any other had advised this remedy in such cases. True, we were aware that authors had recommended it, and reported favorable results from its use in acute and chronic hydrocephalus. But we were not aware that it was advised as an anti-inflammatory remedy in the subjugation of meningitis of a scrofulous character. Our readers are all, doubtless, conscious of the dissimilarity between meningitis and hydrocephalus, though the former often ends fatally in the latter condition. All the authorities that had fallen under our observation had recommended bloodletting, purgatives, mercurials, &c., in meningitis, of whatever form. In the *Philadelphia Medical Examiner* for March, 1853, we had an article upon *scrofulous meningitis,* and its treatment with the iodide of potassium. In the *Northern Lancet* for 1853 we had two articles; and in the Monthly for January, 1856, we had a more elaborate article upon the same subject; while Dr. C. V. W. Burton reports in the Monthly for April, 1857, five cases treated in the same manner. We mention these facts simply because Dr. Coldstream has made no allusion to either of these papers.

Dr. Coldstream says, "In cases of convulsions from teething, which, amongst ill-fed children, living in badly-aired localities, are not unfrequently followed by hydrocephalus, I have used the medicine with much satisfaction." We have often made use of the remedy to allay cerebro-spinal excitement with satisfactory results; but, in the circumstances indicated above, we should expect to render the medication much more efficient by the addition of quinine and asafœtida. Lately we have seen some rather surprising results following the use of the syrup of the hypophosphites under the same circumstances.

*Ovariotomy.*—Before the Pathological Society of London, as per report of proceedings in the *London Lancet* for January, (American reprint,) Dr. Spencer Wells said he had removed *nine* ovarian tumors since the last meeting of the Society. Five of these cases are reported, and the balance promised at a subsequent meeting. Of the five reported, but one died from the effects of the operation. If these are an average of the nine, the results are most satisfactory, and truly remarkable.

These cases and the results are more noteworthy, as most trans-Atlantic surgeons have regarded the operation without favor. Some distinguished French surgeons in particular, have unqualifiedly condemned it. We specify but one instance: M. Velpeau condemns the operation, and says he does not envy his American brethren their practice of ovariotomy. We are certainly glad to learn that Spencer Wells, of London, is operating with such boldness and success. We hope that many more fair unfortunates may find new hope, and a continuance of life in the operation.

In the *Dublin Quarterly Journal*, for Nov., 1859, this same Dr. Wells has an article of much merit upon the subject of ovariotomy, and the means of diminishing the mortality after the operation. Extracts from this article are copied into the January issue of *Braithwaite's Retrospect.* Dr. Wells considers the ligature, and the sloughing of the stump, within the abdominal cavity, the most frequent causes of death, after the operation. To avoid this, he says, "In cases where the peduncle is long, this danger can be avoided by fixing the stump outside the wound; but where the peduncle is short, the écraseur offers evident advantages." In regard to the time of the operation, Dr. Wells makes one remark that perhaps conflicts with the generally received opinion in this country. He says, "It is remarkable that in all the successful cases I have related, the disease was in a very advanced stage; while in the first fatal case it was in a much earlier period of development, and the general health comparatively little injured; but this point again requires more extended inquiry." Dr. Wells is greatly in favor of the use of anæsthetics; the good effects of which he thinks must far outweigh any occasional ill consequences. All the details of the operation and the treatment are given with such minuteness by Dr. Wells, and his results are so satisfactory, that his article should be read by all contemplating the operation. We would gladly make lengthy extracts, but our space, or rather want of space, will not permit, and we shall probably do a better work by thus calling attention to the original paper. Dr. Wells has but little or no confidence in palliative treatment; yet he does not propose rash extirpation. He says, "We do not propose to perform ovariotomy in a case where a woman, if left alone, would probably live in tolerable comfort for several years. We let *her* alone, and do the little we can to increase her comfort. The cases in which we incur the heavy responsibility of performing a dangerous operation, are those in which the patient *must* take her choice, on the one hand, between the risk attendant on such an operation, with a hope of a perfect cure, if all go well; and on the other, a life of suffering, to be terminated, at no distant date, by a miserable death."

Of these cases, Dr. West says, "We come to the sick-chamber day by day, to be idle spectators of a sad ceremony, and leave it humbled by the consciousness of the narrow limits which circumscribe the resources of our art." Dr. Wells says, "We have all seen the poor creatures he so eloquently describes, fading hopelessly away. But the resources of our art are not so limited as he would imply. We may be something more than idle spectators of a death-bed. We have a resource to offer—hazardous, it is true—but one which has in many cases been crowned by a complete and brilliant success." The question of operation is fairly met in the following: "To him who asks, 'How dare I advise an operation we know to be so dangerous?' I answer, How dare you leave the poor woman to die without an effort to save her?"

Before concluding this rather random article on ovariotomy, we wish to refer to one other matter, in, we trust, not inappropriate connection. Many an operation has been commenced and abandoned because of frightful-looking adhesions. The incision has been made, thus exposing the patient to all the risks of peritonitis, and the operation cowardly abandoned; the patient left in the hopeless depression of despair, and the almost certainty of a miserable death. J. Baker Brown, of London—and higher authority cannot well be found—said before the London Medico-Chirurgical Society, he believed them to offer no objection. We quote from a report of the Society's proceedings, in the *London Lancet*, for Feb. 19th, 1859: "The question of adhesions in this disease was one which had led many to consider their existence as opposed to the completion of the operation. He (Dr. Brown) believed, and he was borne out by the great experience of Dr. Clay, that they offered no objection to the operation; indeed, it was doubtful if the peritoneum had not been so thoroughly altered from its normal character, as to be less prone to inflammation on that very account." The adhesions, however, should never be cut, but always torn through.

We conclude with an important and truthful remark of Dr. Brown: "In ovariotomy, a careful after-treatment is of as much importance as a correct diagnosis."

*Diphtheria.*—In the January issue of *Braithwaite's Retrospect,* the views of several distinguished physicians upon this disease are collected from trans-Atlantic journals. We shall briefly give a few of the more important plans of treatment, differing though they do in some particulars. George Bottomley, Esq., Croydon, says, "The treatment I adopted in all the cases under my care was as follows: for *children,*

℞.—Solutionis chlorinii, ℥ss.

Syrupi simplici, ℥ss.

Aquæ distillatæ, ad. ℥vj.

M. Fiat gargarisma sæpe utendum.

℞.—Solutionis chlorinii, gtt. iv.
Syrup. aurantii, ʒj.
Aquæ distillatæ, ad. ℥ss.
M. Fiat haustus 2ndâ quâque horâ sumendus.

The dose was increased according to age. Calomel was given in doses of one grain and upward, according to age. The diet, too, consisted of concentrated jellies, strong beef-tea, wine, &c."

Similar to the above, is the treatment employed by J. C. S. Jennings, Esq., Malmesbury. He says, "The plan I have invariably adopted, regardless of sex or age, or incubation of disease, has been to give an active emetic of antimonial wine, from half an ounce to an ounce, according to age; to freely cauterize the throat with solid nitrate of silver; to have a mustard poultice applied from ear to ear; the feet and legs plunged in a hot bath, and the patient confined to bed."

After the operation of the emetic, a cathartic of calomel and comp. extract colocynth was given, and four hours after the following mixture was ordered:

"℞.—Quinæ disulph., ʒss.

Potassæ chloratis, ʒj.

Acidi hydrochlorici diluti, ℥ss.

Aquæ, ℥viij.

M.—Fiat mistura cujus sumatur par sexta 4tis horis.

A gargle of chlorine solution was directed to be frequently prepared, by impregnating water as much as can be borne with the proto-oxide of chlorine, generated from two parts of chlorate of potass, one of hydrochloric acid and one of water, and the fauces to be sponged out frequently with the same."

C. Swaby Smith, Esq., Burbage, Wiltshire, says, "On first seeing my patient, I apply the strong solution of chlorinated soda to the fauces, and then follow up my treatment by ordering a sinapism to the throat; a gargle, composed of solution of chlorinated soda, two ounces; tincture of myrrh, two drachms; water, six ounces; to be used every half hour, and in cases where the children are too young to gargle, I order the throat to be frequently washed with the same mixture by means of a piece of sponge. Internally, I give to an adult, (of course varying the dose according to my patient's age,) chlorate of potash, two drachms; dilute nitric acid, three drachms; solution of cinchona, (Battley's,) one drachm; water, to six ounces; the sixth part to be taken every two hours. And in cases where there is much pain in the limbs, I generally add a few minims of the tincture of colchicum, which addition has proved decidedly advantageous; the diet to consist of strong beef-tea, port wine, &c.; in short, all the nourishment the patient can take."

Dr. J. S. Bristom, Southwark, says, "It seems to me most important that stimulants, combined with nourishment, should be commenced with early, and should be systematically persisted in." * * * "I, for one, disapprove of the application to the diseased surface of strong caustics and escharoties, and should prefer the employment in all cases of mild detergent gargles."

Thomas Heckstall Smith, Esq., on the contrary, recommends a strong solution of nitrate of silver to the fauces. Besides this, he says, "the treatment consists of bark, ammonia, vegetable acids, abundance of wine, beef-tea, &c., but chiefly gallic acid, in full doses." * * * "But of all the medicines that may present themselves for our choice, there is one far superior, in my experience, to all others, and upon which I indeed chiefly rely: tincture of sesquichloride of iron." This preparation of iron, with wine in large quantities, is also the favorite treatment of Dr. Ranking.

